# 711. A Unique Breath Secondary Metabolite Volatile Signature for the Diagnosis of Histoplasmosis

**DOI:** 10.1093/ofid/ofab466.908

**Published:** 2021-12-04

**Authors:** Armando R Leon, Seena Koshy, Pablo Perez, Sucely Garcia, Nancy Sandoval, Francisco M Marty, Johanna Samayoa, Sophia Koo

**Affiliations:** 1 Brigham and Women's Hospital, Harvard Medical School, Philadelphia, PA; 2 Brigham and Women's Hospital, Boston, MA; 3 Hospital Roosevelt, Guatemala City, Alta Verapaz, Guatemala; 4 Brigham and Women's Hospital, Dana-Farber Cancer Institute, Boston, MA

## Abstract

**Background:**

Histoplasmosis is a common endemic fungal infection in the Americas, causing significant morbidity and mortality, particularly in immunocompromised patients. Existing diagnostic methods are limited in their sensitivity (especially in pulmonary histoplasmosis) and turnaround time.

**Methods:**

We examined prospectively collected breath samples from 84 patients with suspected histoplasmosis 3/2019 - 2/2020 at Hospital Roosevelt (HR; Guatemala City, Guatemala, n = 56) and suspected invasive fungal disease 1/2018 - 10/2019 at Brigham and Women’s Hospital (BWH; Boston, MA, USA, n = 28) using thermal desorption gas chromatography-tandem mass spectrometry (TDU-GC-MS/MS). Patients were evaluated for histoplasmosis and other infections according to the local standard of care – of note, 18/56 patients at HR did not have *Histoplasma* urine antigen testing.

**Results:**

Median age was 44 years, 60 (71%) were male, 23 (27%) had HIV, 15 (18%) had hematologic malignancy. 7 patients were diagnosed with histoplasmosis over the study period (4 at HR, 5 at BWH), with a clinical syndrome + positive *Histoplasma* urine or serum antigen test, with some patients also having yeast forms on tissue biopsy. 3 patients had disseminated and 4 pulmonary histoplasmosis. 4 patients with histoplasmosis had co-infections – 2 tuberculosis (TB), 1 influenza, and 1 *Pneumocystis jirovecii* (PJP) pneumonia. 4 patients were receiving antifungal therapy active against *Histoplasma* at the time of their first breath sample. We found 3 sesquiterpenes: (A) cyperene, (B) 1R,4aR,8aR)-2,5,5,8a-Tetramethyl-4,5,6,7,8,8a-hexahydro-1H-1,4a-methanonaphthalene, and (C) viridiflorol in patients with histoplasmosis, that distinguished these patients from those with other pneumonia (TB, coccidioidomycosis, invasive aspergillosis, mucormycosis, PJP, bacterial pneumonia) with 100% sensitivity and 70% (95% CI 59, 80) specificity.

Figure 1. TDU GC-MS/MS spectral comparison in histoplasmosis vs. the other invasive mycoses aspergillosis or mucormycosis. A: Cyperene; B: (1R,4aR,8aR)-2,5,5,8a Tetramethyl-4,5,6,7,8,8a-hexahydro-1H-1,4a-methanonaphthalene; C: viridiflorol; D: 1H-Indene, 2,3,3a,4-tetrahydro-3,3a,6-trimethyl-1-(1-methylethyl)-; E: β-funebrene; F: trans-α-bergamotene; G: eremophilene; H: spathulenol; I: cedrene; J: cedranoxide, 8,14-

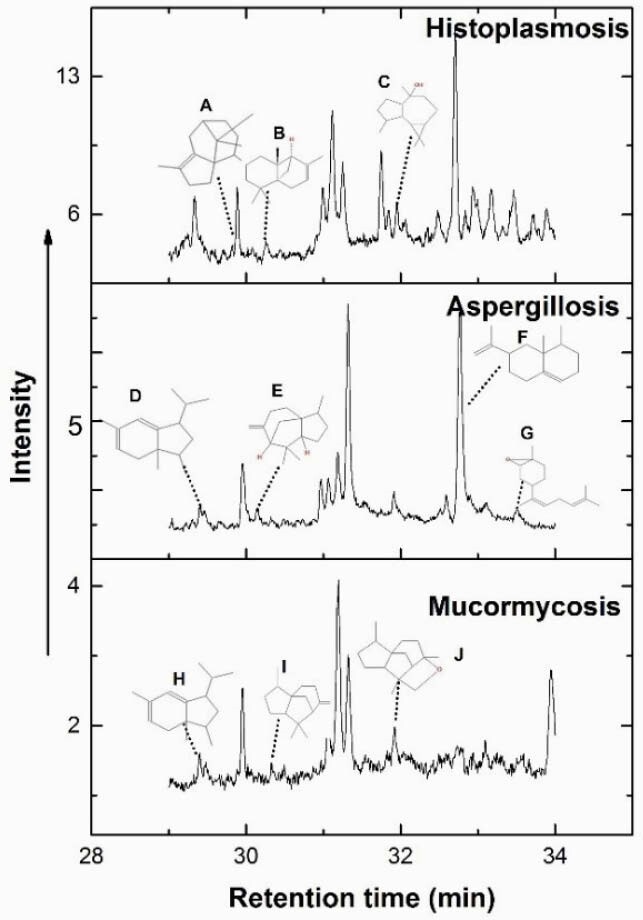

**Conclusion:**

**Conclusion:** Patients with histoplasmosis have a unique secondary metabolite breath signature that can be used for the noninvasive diagnosis of pulmonary and disseminated histoplasmosis. Many patients in this cohort did not undergo urine antigen testing or other diagnostic workup for histoplasmosis, which may have affected our specificity estimates.

**Disclosures:**

**Francisco M. Marty, MD**, **SCYNEXIS, Inc.** (Scientific Research Study Investigator)

